# AHR is a master regulator of diverse pathways in endogenous metabolism

**DOI:** 10.1038/s41598-022-20572-2

**Published:** 2022-10-05

**Authors:** Jeffry C. Granados, Kian Falah, Imhoi Koo, Ethan W. Morgan, Gary H. Perdew, Andrew D. Patterson, Neema Jamshidi, Sanjay K. Nigam

**Affiliations:** 1 Department of Bioengineering, University of California San Diego, La Jolla, CA 92093 USA; 2 Departments of Biology, University of California San Diego, La Jolla, CA 92093 USA; 3 Department of Veterinary and Biomedical Sciences, Center for Molecular Toxicology and Carcinogenesis, The Pennsylvania State University, University Park, PA 16802 USA; 4 Department of Biochemistry and Molecular Biology, The Pennsylvania State University, State College, PA 16801 USA; 5 Department of Radiological Sciences, University of California Los Angeles, Los Angeles, CA 90095 USA; 6 Department of Pediatrics, University of California San Diego, La Jolla, CA 92093 USA; 7 Department of Medicine (Nephrology), University of California San Diego, La Jolla, CA 92093 USA

**Keywords:** Biochemical networks, Transcription, Metabolomics, Biochemical reaction networks

## Abstract

The aryl hydrocarbon receptor (AHR) is a transcription factor with roles in detoxification, development, immune response, chronic kidney disease and other syndromes. It regulates the expression of drug transporters and drug metabolizing enzymes in a proposed Remote Sensing and Signaling Network involved in inter-organ communication via metabolites and signaling molecules. Here, we use integrated omics approaches to analyze its contributions to metabolism across multiple scales from the organ to the organelle. Global metabolomics analysis of *Ahr*^−/−^ mice revealed the role of AHR in the regulation of 290 metabolites involved in many biochemical pathways affecting fatty acids, bile acids, gut microbiome products, antioxidants, choline derivatives, and uremic toxins. Chemoinformatics analysis suggest that AHR plays a role in determining the hydrophobicity of metabolites and perhaps their transporter-mediated movement into and out of tissues. Of known AHR ligands, indolepropionate was the only significantly altered molecule, and it activated AHR in both human and murine cells. To gain a deeper biological understanding of AHR, we employed genome scale metabolic reconstruction to integrate knockout transcriptomics and metabolomics data, which indicated a role for AHR in regulation of organic acids and redox state. Together, the results indicate a central role of AHR in metabolism and signaling between multiple organs and across multiple scales.

## Introduction

The aryl hydrocarbon receptor (AHR) is a ligand-activated transcription factor best known for its role in sensing xenobiotics and regulating the expression of genes involved in xenobiotic metabolism, including drug transporters and drug metabolizing enzymes (DMEs)^1^. In addition, AHR has been shown to contribute to development and immune response, as well as metabolite signaling, suggesting an important role in endogenous metabolism^2–4^. Consistent with its function in these important physiological processes, AHR is widely expressed, with relatively high expression in multiple organs. According to the Remote Sensing and Signaling Theory, ligand-activated transcriptional regulators, such as AHR, play a key role in remote inter-organ communication via small molecules through their regulation of drug transporters and DMEs across multiple organs^5–8^. Together these transporters and DMEs modulate levels of small molecules in organs, cells, and body fluids. That some of the transported small molecules activate AHR creates the potential for homeostatic feedback loops.

For AHR to exert its transcriptional effects, it must first bind to a ligand and form a heterodimer with ARNT^9^. AHR has traditionally been studied with respect to its high affinity for halogenated aromatic hydrocarbon or polycyclic aromatic hydrocarbon ligands^10^. One of the best characterized ligands is the ubiquitous pollutant 2,3,7,8-tetrachlorodibenzo-*p*-dioxin (TCDD), which has been shown to alter detoxification, immune response, and development through its binding to AHR in cellular assays and *in vivo*^11–15^. Genetically modified mouse models, including *Ahr*^−/−^ mice coupled with ligand exposure models, have elucidated other potential endogenous roles of AHR. A previous study revealed that *Ahr*^−/−^ mice exhibited an increase in serum bile acids (BAs) compared to *Ahr*^+/+^ mice^16^. The deletion of *Ahr* also altered the expression of important hepatic transporter genes, with downregulation of *Oatp1a1*, *Oatp1b2*, *Oatp2b1*, and *Oct1*^20^ and upregulation of *Cyp7a1*—the initial and rate limiting enzyme in the traditional BA generation pathway starting from cholesterol^16^. Furthermore, exposure of 2,3,7,8-tetrachlorodibenzofuran (TCDF), a potent AHR ligand, in *Ahr*^+/+^ mice led to the disruption of lipid and carbohydrate metabolites in the serum, whereas the same exposure in *Ahr*^−/−^ mice resulted in no significant changes in glucose or lipid levels^17^.

TCDD and TCDF are the prototypical activators of AHR, but dozens of other synthetic and natural agonists and antagonists with varying affinities have been identified^18^. While many *in vitro* and *in vivo* studies have analyzed the effects of TCDD/TCDF activation, there has also been interest in how different ligands might impact the transcriptional activity of AHR. For example, the polyphenolic AHR ligand quercetin was presented to human hepatocytes and increased expression of PON1 and CYP1A1, while the traditional AHR agonist TCDD only induced CYP1A1 expression^19^. These distinct changes based on ligand structure and affinity could be important because of the many endogenous compounds, such as tryptophan metabolites, that are relatively weak ligands for AHR and may contribute to basal levels of AHR activation^20–22^. The levels of these circulating compounds can be altered by disease states, such as chronic kidney disease (CKD)^7^. In CKD, loss of renal function leads to the accumulation of solutes that are typically cleared by the kidneys. Several of these uremic solutes toxins, including indoxyl sulfate, kynurenine, and kynurenate, are also AHR ligands^23^. Indoxyl sulfate is among the best characterized uremic toxins and has been shown to accumulate in the blood of CKD patients and initiate chronic AHR activity in other tissues besides the kidney^24,25^. The increased concentration of these solutes may partly explain some of the widespread proinflammatory states and cardiovascular disease seen in patients with CKD. Though these compounds are primarily known as uremic toxins, they may have beneficial effects that act via AHR signaling, and activate the proposed role of AHR in metabolite-mediated remote sensing and signaling between organs and organisms^7,25,26^.

These studies indicate the need for a more comprehensive understanding of the role of AHR in endogenous physiology. While most research emphasizes the transcriptional role of AHR in different tissues, we focused on the alterations in a large and diverse set of serum metabolites, as well the metabolic pathways they participate in. Global metabolomics of *Ahr*^−/−^ and *Ahr*^+/+^ mice revealed hundreds of metabolites had their circulating levels significantly altered by loss of *Ahr.* These metabolites are involved in fatty acid metabolism, choline metabolism, sphingolipid synthesis, bile acid metabolism, uremic toxin generation among several other biochemical pathways and functional clusters. To provide a multiscale portrait of altered metabolism in the *Ahr*^−/−^ mice, we also integrated serum metabolomics and cellular transcriptomic data to develop systems biology metabolic reconstructions using Recon3D that mapped the various metabolic pathways, from the organ level to the organellar level, that are regulated by AHR. The reconstruction revealed important roles for AHR in nitrogen elimination, peroxisomal fatty acid oxidation and mitochondrial metabolism, indicating that AHR exerts important metabolic effects across multiple scales (e.g., organism, organ, cell, organelle).

## Results

### Deletion of *Ahr* expression alters 290 of 965 measured metabolites participating in numerous metabolic pathways

**Figure 1:**
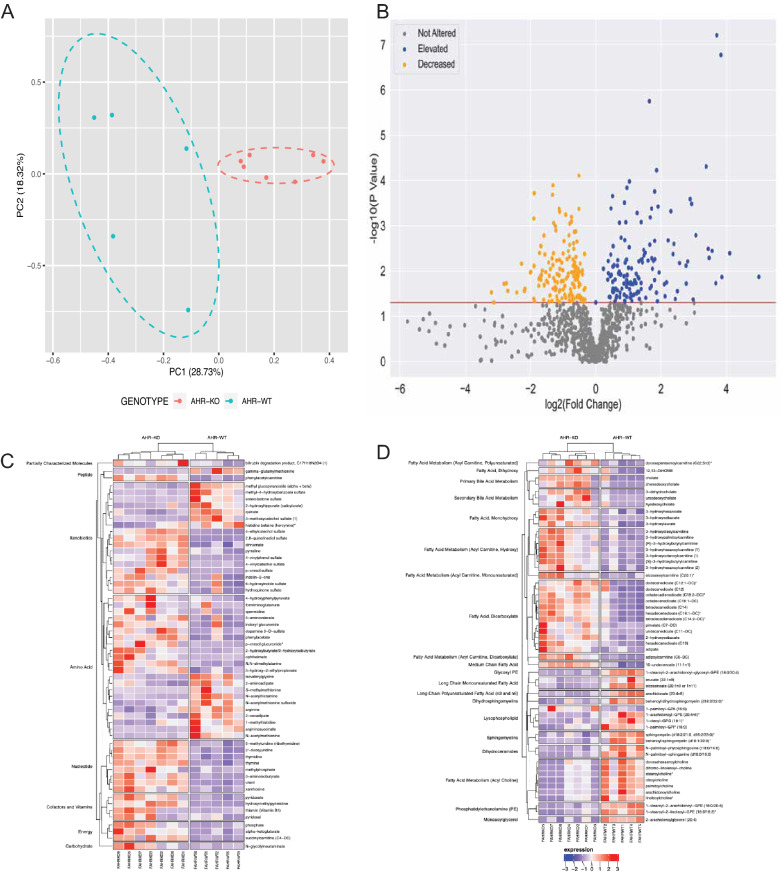
Loss of *Ahr* has a major impact on the serum metabolome of mice. (**A**) Principal component analysis reveals separation between the *Ahr*^−/−^ and *Ahr*^+/+^ mice. (**B**) Of the 965 surveyed metabolites, 290 were significantly altered (*p* < 0.05), with 138 elevated and 152 decreased. (**C**) Heatmap of the most significantly altered non-lipids (Partially Characterized Molecules, Peptides, Xenobiotics, Amino Acids, Nucleotides, Cofactors and Vitamins, Energy, and Carbohydrate superpathways) show the differences between *Ahr*^−/−^ (n = 7) and *Ahr*^+/+^ (n = 5). (**D**) The Lipid superpathway, the largest measured superpathway, also has several altered metabolites. Heatmaps were generated using the ComplexHeatmap in R software environment.

The broad range of genes, including drug transporters and drug metabolizing enzymes, in multiple tissues regulated by AHR suggests that loss of the gene would lead to changes in the expression of proteins involved in the absorption, distribution, metabolism, and excretion (ADME) of small molecules and/or a proposed Remote Sensing and Signaling Network^27^. Since many of these transporters and enzymes are known to participate in local and systemic metabolism, we expected alterations in the circulating levels of multiple metabolites and signaling molecules. To test this hypothesis, serum from female *Ahr*^−/−^ (n = 5) and *Ahr*^+/+^ mice (n = 7) was globally metabolically profiled. Overall, 965 metabolites were detected in the samples, including metabolites that had been incompletely characterized. These metabolites covered 109 unique biochemical subpathways (e.g., primary bile acid metabolism, tryptophan metabolism, benzoate metabolism). The *Ahr*^−/−^ and *Ahr*^+/+^ mice were clearly separable by genotype with principal component analysis (Fig. 1A). In total, 138 metabolites were significantly elevated (*p* < 0.05, fold change > 1) and 152 were significantly decreased (*p* < 0.05, fold change < 1), with 55 more trending towards significant elevation (0.05 < *p* < 0.10, fold change > 1) and 49 more trending towards significant decreases (0.05 < *p* < 0.10, fold change < 1) (Fig. 1B, Supplementary Table S1). Selected elevated and decreased metabolites representing a wide array of biochemical pathways with accompanying fold changes, *p*-values, and q-values are shown in Tables 1 and 2, respectively. The data were also separated into non-lipids and lipids to show the distinct differences in specific metabolites (Fig. 1C,D).

**Table 1:** Elevated metabolites in the *Ahr* knockout cover a wide range of biochemical pathways, with many showing fold changes greater than 2.

Metabolite	Pubchem ID	Superpathway	Subpathway	Fold change	*p*-value	q-value
Cholate	221493	Lipid	Primary bile acid metabolism	14.60	0.0137	0.0310
Hydroxymethylpyrimidine	777	Cofactors and vitamins	Thiamine metabolism	13.09	0.0000	0.0000
Beta-muricholate	5283853	Lipid	Primary bile acid metabolism	12.85	0.0186	0.0350
chenodeoxycholate	10133	Lipid	Primary bile acid metabolism	11.85	0.0036	0.0217
3-dehydrocholate	159655	Lipid	Secondary bile acid metabolism	11.15	0.0051	0.0244
p-cresol glucuronide*	154035	Amino acid	Tyrosine metabolism	10.94	0.0032	0.0204
4-vinylphenol sulfate	6426766	Xenobiotics	Benzoate metabolism	10.47	0.0000	0.0042
phenylacetylcarnitine	101724840	Peptide	Acetylated peptides	7.66	0.0003	0.0071
Ophthalmate	7018721	Amino acid	Glutathione metabolism	6.86	0.0189	0.0351
Azelate (C9-DC)	2266	Lipid	Fatty acid, dicarboxylate	6.84	0.0077	0.0262
Spermidine	1102	Amino acid	Polyamine metabolism	3.51	0.0158	0.0332

**Table 2:** Decreased metabolites in the *Ahr* knockout cover a wide range of biochemical pathways and fold changes.

Metabolite	Pubchem ID	Superpathway	Subpathway	Fold change	*p*-value	q-value
Cystine	67678	Amino acid	Methionine, cysteine, SAM and taurine metabolism	0.22	0.0245	0.0402
Mead acid (20:3n9)	5312531	Lipid	Long chain polyunsaturated fatty acid (n3 and n6)	0.27	0.0408	0.0536
Argininosuccinate	60150382	Amino acid	Urea cycle; arginine and proline metabolism	0.31	0.0028	0.0187
Linoleoylcholine*	53481656	Lipid	Fatty acid metabolism (acyl choline)	0.31	0.0123	0.0301
Arginine	6322	Amino acid	Urea cycle; arginine and proline metabolism	0.34	0.0106	0.0294
2-hydroxyhippurate (salicylurate)	10253	Xenobiotics	Benzoate metabolism	0.37	0.0128	0.0303
Arachidonate (20:4n6)	444899	Lipid	Long chain polyunsaturated fatty acid (n3 and n6)	0.42	0.0166	0.0340
Xanthurenate	5699	Amino acid	Tryptophan metabolism	0.56	0.0036	0.0217
Kynurenate	3845	Amino acid	Tryptophan metabolism	0.69	0.0106	0.0294

**Figure 2:**
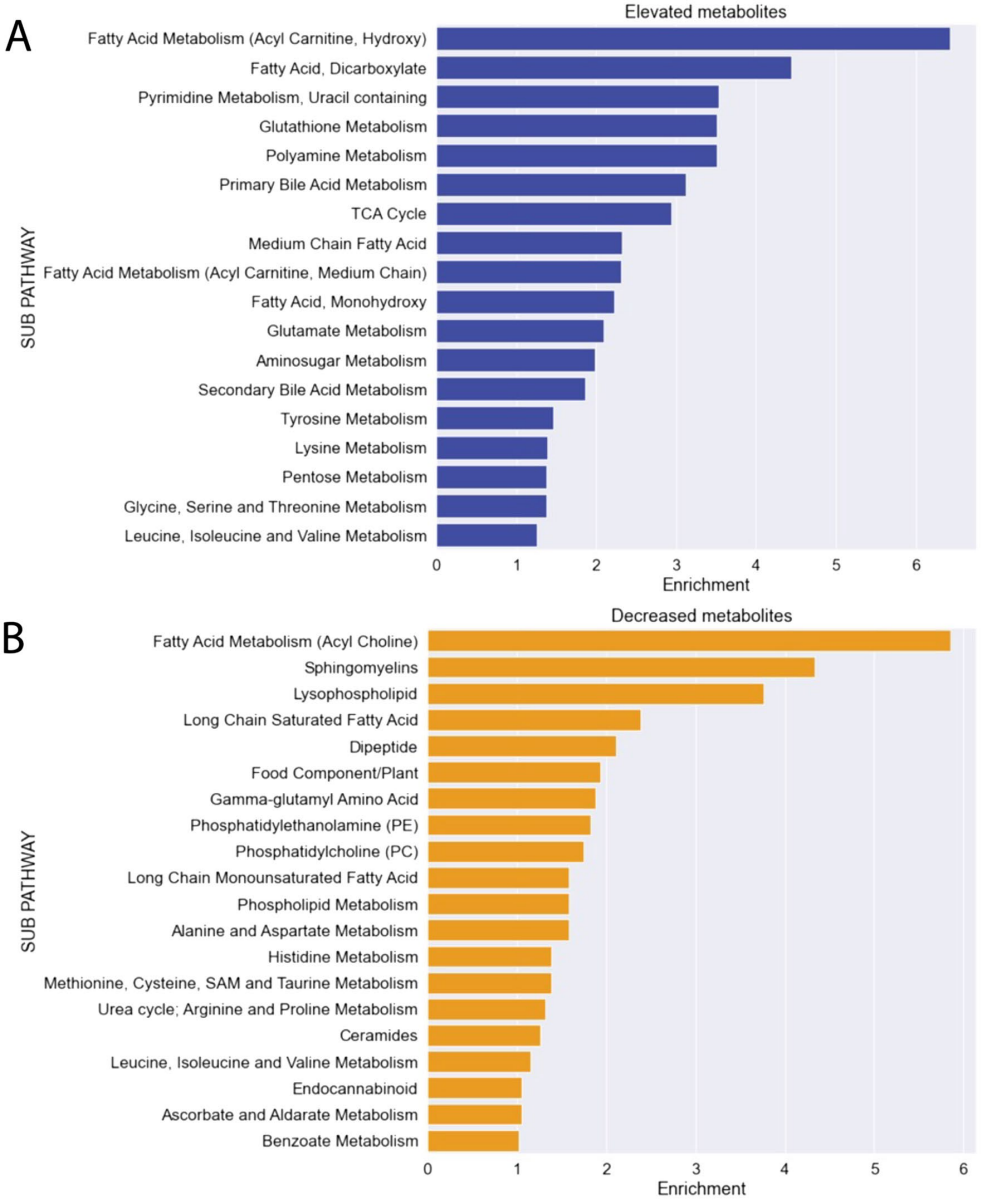
Pathway enrichment revealed that loss of AHR alters numerous, distinct biochemical subpathways. (**A**) The fatty acid metabolism (acyl carnitine, hydroxy), glutathione metabolism, and primary bile acid metabolism subpathways were the most enriched of the 18 subpathways with elevated metabolites. (**B**) Other aspects of fatty acid metabolism (acyl choline), sphingomyelins, and lysophospholipids subpathways were the most enriched of the 20 subpathways with decreased metabolites.

As AHR is a transcriptional regulator that modulates the expression of a variety of genes across many tissues and does not itself directly contribute to the ADME of metabolites, there is no straightforward mechanism for the increases or decreases in serum metabolites (except through the effects of AHR on transporter and enzyme expression). However, it is possible that metabolites ultimately regulated by AHR are involved in certain biological processes. We first focused on the Metabolon-designated subpathways, which can be structurally related but are mainly defined based on their role in the relevant biochemistry. For example, all metabolites derivative of tryptophan are grouped into the tryptophan metabolism subpathway. Through this biochemically focused lens, we identified 18 subpathways were enriched for significantly elevated metabolites (*p* < 0.05, fold change > 1), including fatty acid metabolism (designated by Metabolon as acyl carnitine, hydroxy), primary bile acid metabolism, and several others (Fig. 2A). Among the 20 subpathways with an enrichment value over 1 for significantly decreased metabolites (*p* < 0.05, log2 of fold change < 1) were other aspects of fatty acid metabolism designated by Metabolon as fatty acid metabolism (acyl choline), sphingomyelins, and lysophospholipids (Fig. 2B).

**Figure 3:**
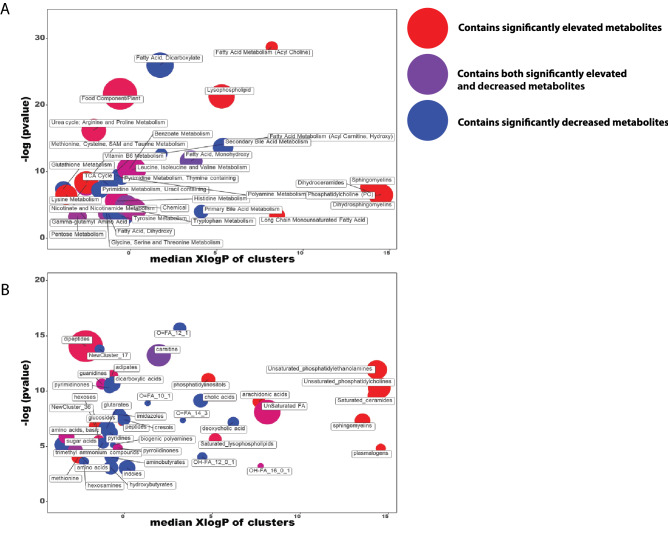
Chemistry-driven functional clustering yields different results from biochemically defined subpathway enrichment. (**A**) ChemRICH analysis of all the significantly altered compounds under *Ahr*^−/−^ versus *Ahr*^+/+^ conditions with the Metabolon-defined subpathways echoes the results of the pathway enrichment, with a combination of elevated and decreased pathways being statistically altered. (**B**) Novel functional clusters were generated for each metabolite with a known chemical structure based on similarity to other measured metabolites. The most altered pathways differed from those defined by Metabolon’s biochemically-based subpathways, revealing that dipeptides, carnitine derivatives, and dicarboxylic acids were the most significantly impacted metabolic functional clusters. In both panels, the size of each point represents the number of compounds within the subpathway/functional cluster, the color represents the proportion of significantly increased/decreased compounds within the cluster, where blue points have more decreased compounds, purple points have elevated and decreased compounds, and red points have more elevated compounds, with intermediate colors between these representing different ratios. The x-axis is the median XlogP (relative lipophilicity based on the enhanced atomic partition coefficient) of all the compounds within the cluster, and the y-axis is the -log of the *p*-value, as calculated by the Kolmogorov–Smirnov test.

While subpathway enrichment calculations were useful in interpreting the results from a metabolic perspective, annotation of the chemical space with current mass spectrometry approaches is still evolving. Certain classes of metabolites may be over-represented or absent in the set of measured metabolites corresponding to a particular subpathway, possibly leading to an inaccurate picture of the relative importance of various subpathways. Furthermore, certain broader pathways contain metabolites that may not be represented by conventional biochemical pathways and/or poorly understood from the viewpoint of their importance in systemic biochemistry. For example, metabolites mainly stemming from the diet are grouped into the food/component plant subpathway, one of the largest subpathways, with 63 unique metabolites in it. To overcome these potential limitations and provide a more chemoinformatic view, we focused on the structural similarities between metabolites. We generated functional clusters for each metabolite with an associated Pubchem ID (794 metabolites) using the ChemRICH web-based tool, which uses the chemical structure of each metabolite to identify similar metabolites^28^. Each metabolite was grouped into one of 179 novel functional clusters based on the Medical Subject Headings (MeSH) maintained by the National Institute of Health (NIH) (Supplementary Table S2). To compare these chemically driven results to our biochemically-oriented results, we first performed statistical analysis on the initial Metabolon-designated subpathways and noted some differences between the subpathway enrichment results and the ChemRICH results, namely that the food component/plant (one of the most chemically diverse pathways) and lysophospholipid (one of the least chemically diverse pathways) subpathways were the 3rd and 4th most altered, respectively (Fig. 3A). However, using the chemically driven approach, we found that the most significantly altered functional clusters were long chain dicarboxylic acids (O=FA_12_1), dipeptides, conjugated carnitines, and carnitine-related pathways (Fig. 3B). Together, these two approaches (pathway driven subpathway enrichment and chemically-driven functional clusters) complement each other by focusing on different perspectives of small molecule metabolites.

### Metabolites decreased in the serum of *Ahr*^−/−^ mice are characterized by greater hydrophobicity and molecular weight

**Figure 4:**
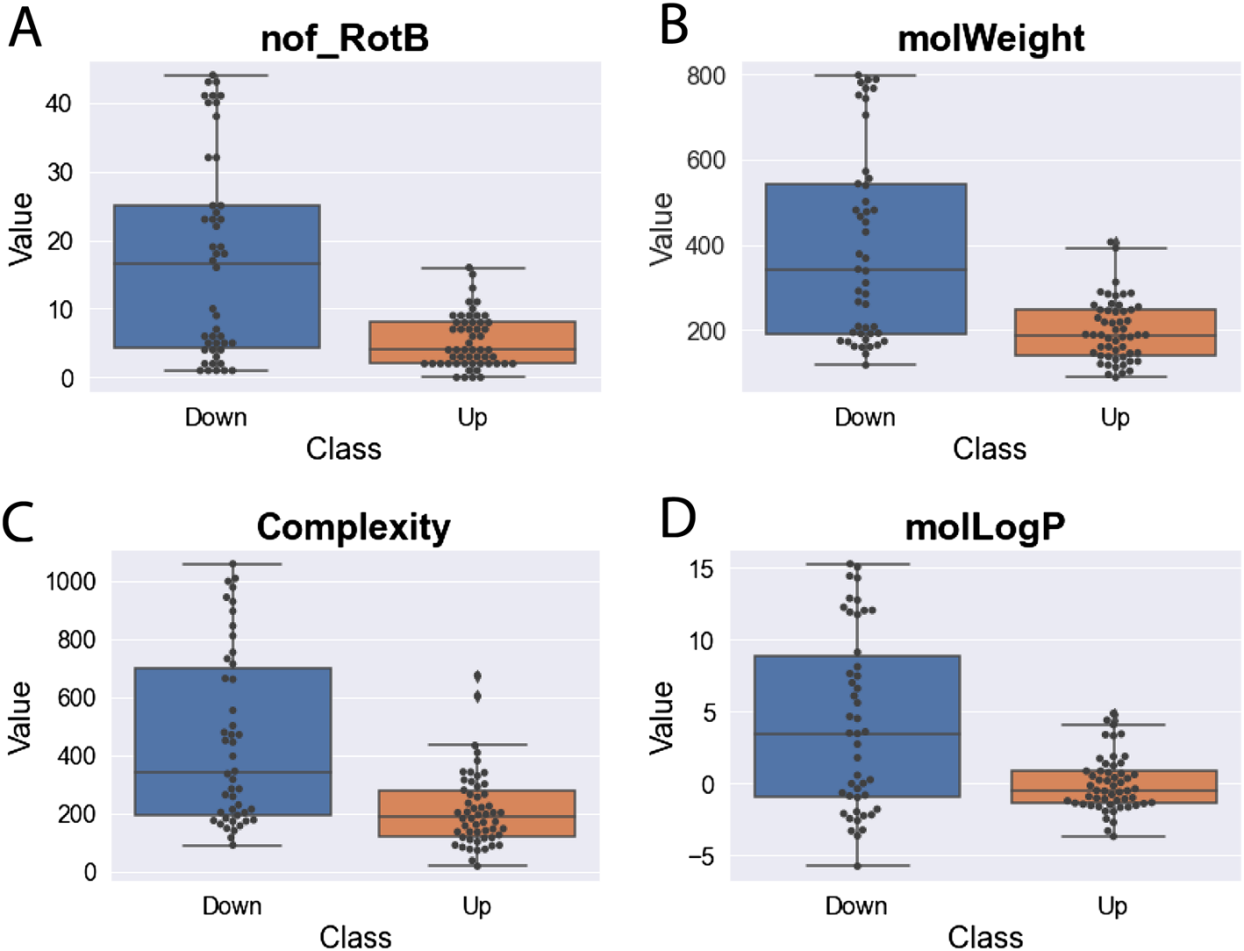
Elevated (n = 54) and decreased metabolites (n = 46) are largely separable by four physicochemical properties. (**A**) Water-octanol partition coefficient (molLogP) is higher in the decreased compounds, indicating more hydrophobic compounds. (**B**) Molecular weight (molWeight) is higher in decreased compounds. (**C**) Complexity, as calculated by Pubchem, is higher in decreased compounds. (**D**) Number of rotatable bonds (nof_RotB) is higher in decreased compounds, indicating that the decreased compounds are less rigid. Box plots include the median (central line), lower quartile (lower limit of box), upper quartile (upper limit of box), max value (upper limit of whisker), and minimum value (lower limit of whisker). Each metabolite in this set is represented by an individual point.

We noted that many subpathways/functional clusters that were significantly altered tended to contain only elevated or only decreased compounds, indicating that AHR affected the pathways as a whole and not a specific reaction between compounds in the same subpathways/functional clusters. Thus, we aimed to analyze the physicochemical properties that separated elevated and decreased metabolites. We calculated 77 molecular properties for the metabolites in the dataset with chemical structures, using ICM Molsoft Pro. To maximize differences in structural features, we used a stricter *p*-value threshold (*p* < 0.01) to assess statistical significance. Using this more stringent criterion, 54 metabolites were considered elevated, and 46 metabolites were considered decreased, representing nearly even subsets. Univariate and other analyses in Orange data mining software^29^ revealed that water-octanol partition coefficient (molLogP), molecular weight (molWeight), complexity, and number of rotatable bonds (nof_RotB) were the molecular properties that best separated the elevated and decreased compounds (Fig. 4). This suggests AHR may be an important regulator of proteins, like conjugating enzymes, that increase the hydrophilicity of a compound.

### Indolepropionate is the only known AHR ligand significantly elevated in vivo

Our primary focus was on the metabolites that were altered by loss of *Ahr*, but we also aimed to explore the effect on the circulating levels of AHR ligands, as there may exist feedback loops involving AHR activation that keep these levels constant. AHR ligands can partially mediate their own levels by changing the expression patterns of proteins that act upon them, as in the case of indoxyl sulfate through OAT1^30^. Loss of AHR would potentially lead to disruption of these feedback loops, which could result in metabolic consequences. In the absence of xenobiotics, it might be expected that the endogenous ligands of AHR, like tryptophan derivatives, activate AHR transcriptional regulation in the wild type mice, but not the *Ahr*^−/−^ mice.

**Figure 5:**
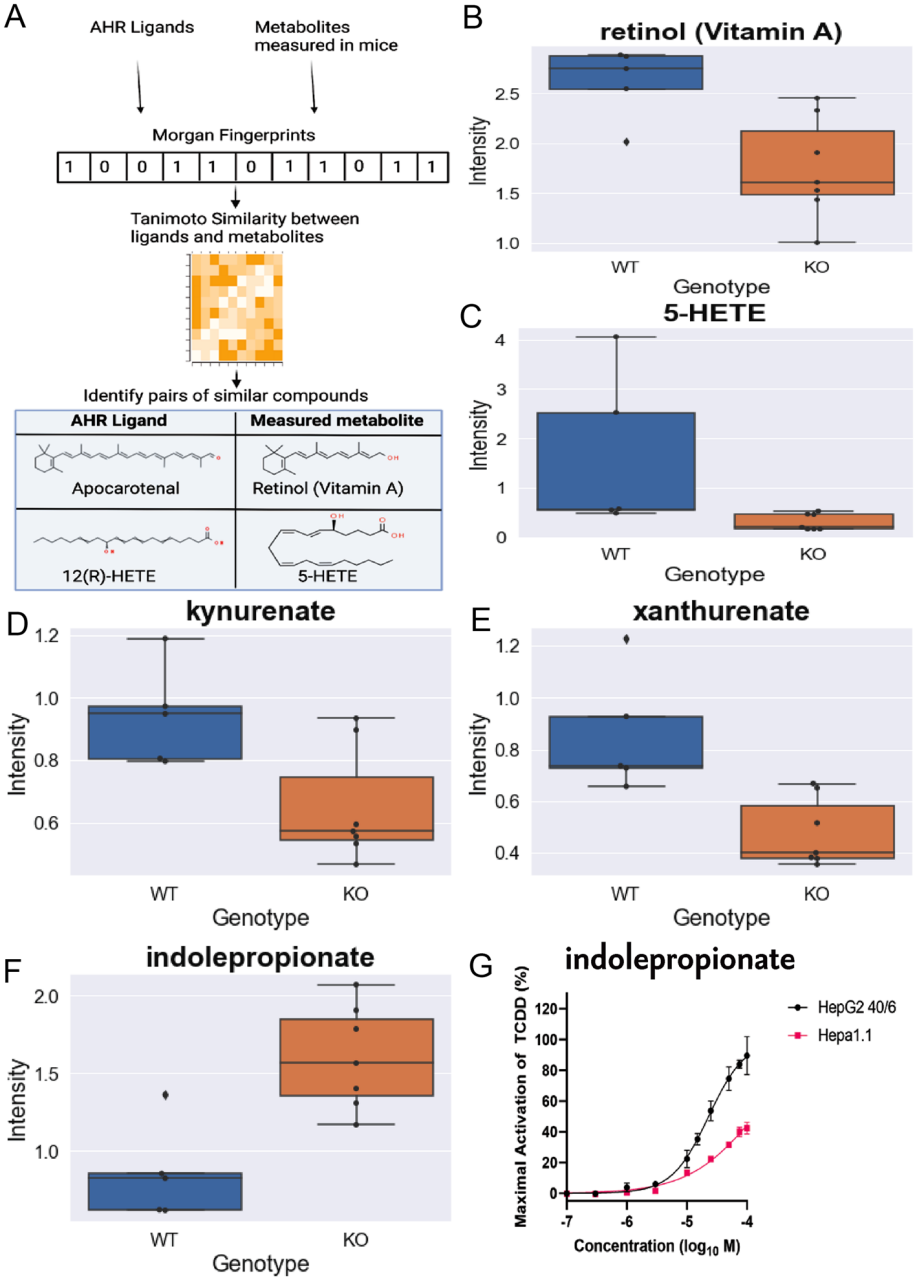
Five AHR ligands or compounds chemically similar to ligands are significantly altered in the serum of *Ahr*^−/−^ mice (n = 7) compared to *Ahr*^+/+^ mice (n = 5). (**A**) Known AHR ligands were chemically compared to the measured metabolites using Tanimoto index to identify similar compounds. Five of the 25 measured AHR ligands and structurally related metabolites were among the 290 significantly altered metabolites. (**B**) Retinol (Vitamin A), structurally similar to apocaretanol was significantly decreased in the serum. (**C**) 5-HETE, similar to the AHR ligand 12(R)-HETE, was significantly decreased in the serum. (**D**) Kynurenate was significantly decreased in the serum. (**E**) Xanthurenate was significantly decreased in the serum. (**F**) Indolepropionate was the only AHR ligand that was elevated in the serum. Box plots include the median (central line), lower quartile (lower limit of box), upper quartile (upper limit of box), max value (upper limit of whisker), and minimum value (lower limit of whisker). Each metabolite in this set is represented by an individual point. (**G**) Indole-3-propionic acid was assessed for its ability to activate both human and murine AHR at ten biologically relevant doses (100 nM, 300 nM, 1, 3, 10, 15, 25, 50, 75, 100 μM final concentrations). TCDD (10 nM final concentration) was used to determine maximal activation of AHR, and the presented values were normalized as a percentage of TCDD activation. Each data point represents the mean ± S.D. of three biological replicates.

We curated a list of synthetic and endogenous compounds from the literature that are known to interact with AHR (antagonists and agonists) or activate AHR-reporter genes (Supplementary Table S3). Since many of these compounds were not measured in our metabolomics experiment, we also calculated the chemical similarity between the known AHR-interacting molecules and the metabolites measured *in vivo* to see whether metabolites that are structurally similar to known ligands are impacted in the *Ahr*^−/−^ mice (Fig. 5A). Of the 25 compounds that are either AHR ligands or structurally similar to AHR ligands (Tanimoto similarity index > 0.6), five were significantly altered, with three directly measured (kynurenate, xanthurenate, indolepropionate) and two being considered structurally similar to known ligands. 5-HETE was similar to 12(R)-HETE (Tanimoto coefficient 0.61) and retinol (Vitamin A) was similar to apocarotenal (Tanimoto coefficient 0.71). Thus, only 5 of 290 altered metabolites were identical to, or chemically similar to, known AHR ligands (Fig. 5). The relatively low number does not reveal obvious feedback mechanisms where loss of AHR may alter the levels of its ligands, though this interpretation may change once more AHR ligands are discovered. Of the 5 altered compounds, 4 were significantly decreased (Fig. 5B–E). A single known AHR ligand, indolepropionate, was elevated in the *Ahr*^−/−^ mice (Fig. 5F).

Previous results have indicated that there are differences between how murine and human AHR respond to different ligands. Thus, it is possible that the proposed feedback loops involving AHR and its ligands are weaker in mice than in humans. To address this, reporter assays were performed with a Hepa1.1 cell line, derived from a murine hepatoma, and a HepG2 40/6 cell line, derived from a human hepatoma, both of which possess an integrated copy of a DRE-driven luciferase reporter construct. The reporter cell lines were used to compare potential AHR transcriptional activity between the two species when exposed to indolepropionate. Generated EC_50_ values indicate significantly different AHR activation by these three metabolites in a species-dependent manner (*p* < 0.0005) (Fig. 5G). Nonetheless, it appears that for indolepropionate, there may exist a feedback loop in wildtype mice where AHR lowers circulating levels.

### Multiscale integration of metabolomic and human transcriptomic data via genome scale metabolic reconstruction

To better understand the potential causes for the metabolomic changes in the serum, we analyzed transcriptomic datasets that described the function of AHR with activation, inhibition, knockout, or some combination of these. We found studies in the murine liver, murine kidney and MCF7 cells that demonstrated changes in gene expression due to disruption of AHR regulation (Supplementary Table S4). Due to the major impact that loss of *Ahr* had on the serum metabolome, we initially focused on drug transporters and drug metabolizing enzymes, as these proteins are known to play pivotal roles in the ADME of small molecules in the blood. We noted altered expression of several key transporters (*Slc22a6, Abcg2, Abcc4*, etc.) and enzymes (*Akr*, *Adh*, *Cyp*, *Sult* genes) in the knockout kidney and liver. We also analyzed MCF7 cells in which *AHR* had been knocked down to understand the role of AHR in a genetically homogenous cell line. While this transcriptomics data was generally consistent with that obtained from knockout liver and kidney, the cell line knockdown of *AHR* more clearly demonstrated downregulation of genes involved in metabolic processes for both exogenous and endogenous compounds, with two of the most enriched processes being flavonoid and phase II metabolism. *AHR* knockdown revealed a significant increase in processes involved in mitotic replication, such as DNA repair checkpoints and cytokinesis, which is consistent with AHR’s known role in development, immune response, and injury repair.

We then integrated transcriptomic data from relevant datasets and our serum metabolomics data to develop genome-scale metabolic models (GSMM) for *Ahr*^−/−^ and *Ahr*^+/+^ mice with Recon3D. Recon3D maps altered genes to a large metabolic map to predict how transcriptomic changes impact metabolic function. These types of multi-omics, multi-scale reconstructions have been shown to go beyond conventional pathway analysis by defining the metabolic impact of the loss of, for example, drug transporter genes^31–33^. Here, GSMMs were constructed to help interpret the metabolic alterations resulting from gene expression changes and serum metabolomic alterations and emphasize other processes within cells that are affected.

**Figure 6:**
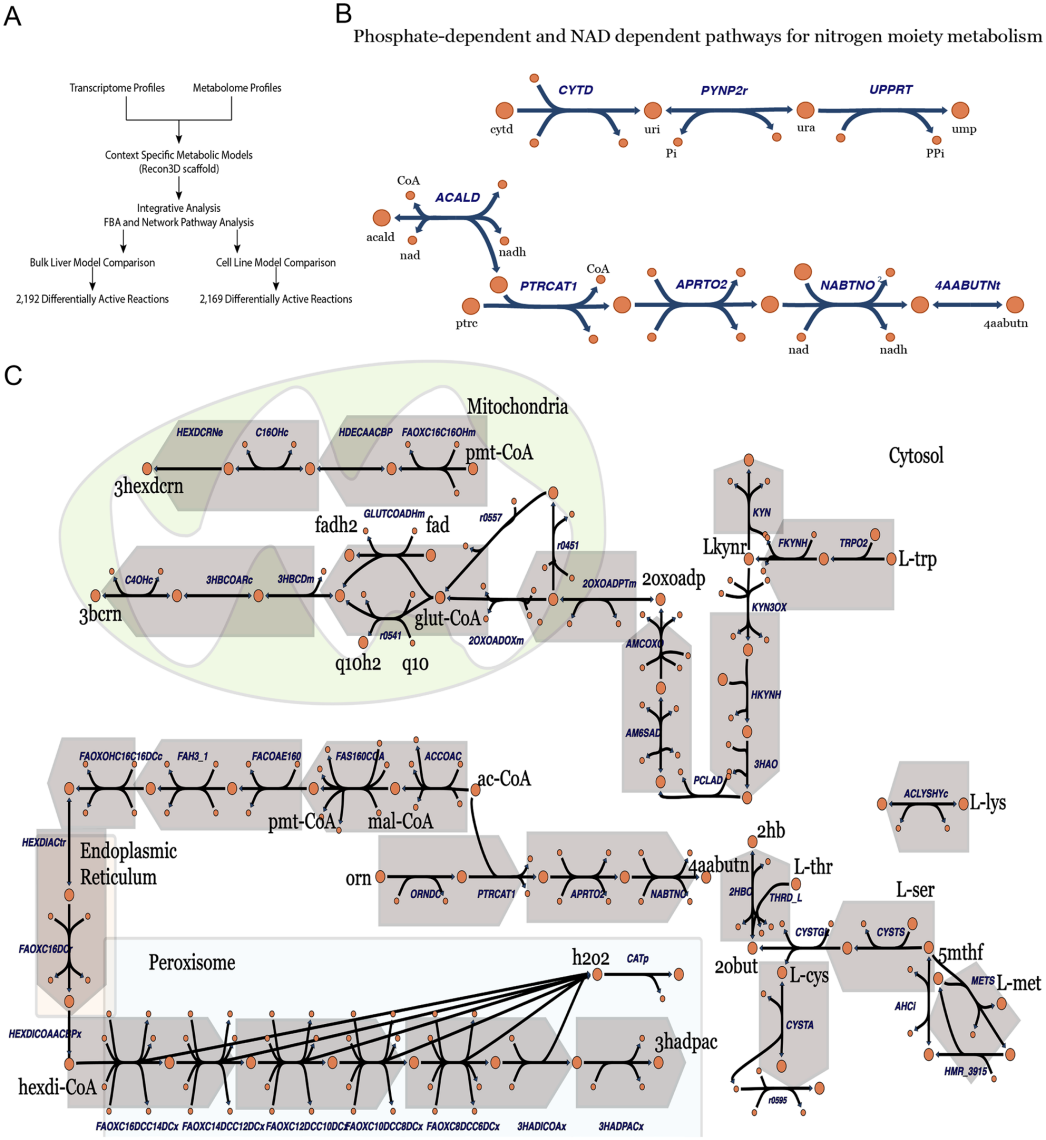
Metabolic reconstructions combining transcriptomic data with serum metabolomics data reveal roles for AHR in cellular and subcellular processes. (**A**) Schematic describing integration of multi-omics data through metabolic reconstruction. Serum metabolomics from the knockout mice and transcriptomic data from two different experiments (*Ahr*^−/−^ liver and *AHR* knockdown MCF7 cells) were combined using Recon3D to develop distinct genome scale metabolic reconstructions for the *Ahr*^−/−^ and *Ahr*^+/+^ mice. These models were then compared to determine the number of differentially active reactions. (**B**) Functional pathway analysis of metabolic alterations in AHR wildtype versus knockout in mouse liver. Nitrogen handling was noted to be a prominent metabolic process involved in the enzymatic reaction fluxes that were increased in knockout relative to wildtype conditions. Two pathways consisting of reactions that were differentially active based on flux simulations are depicted. The top pathway is dependent on transfer of ortho- and pyro-phosphate. The lower pathway involves NAD/NADH cofactors and CoA. Reactions are provided in Supplemental Table S5. Metabolite abbreviations: ura: uracil, acald: acetaldehyde, uri: uridine, duri: deoxyuridine, ptr: putrescine, 4aabutn: 4-acetamidobutanoate, Pi: orthophosphate, PPi: pyrophosphate, CoA: coenzyme A. (**C**) Functional pathway analysis of metabolic alterations in AHR wildtype versus knockout. Selected, representative reactions highlighting the organelle-specific pathways with predicted increased fluxes in *AHR* knockdown. Peroxisomal fatty acid oxidation with associated generation of hydrogen peroxide (and reduction by catalase) was increased for multiple fatty acids. Degradation of multiple amino acids, including tryptophan, converge on the increased production of multiple organic acids. Increased mitochondrial elimination pathways include glutaryl-CoA that is ultimately converted to 3-hydroxybutyrylcarnitine before being transported out of the cell. Most biosynthetic and degradation reactions deplete the NADPH and NADH cofactor pools, however, some of the mitochondrial processes recharge the FADH2 and Q102 pools. Reactions are provided in Supplemental Table S6. Metabolite abbreviations (black text): L-tyr: L tyrosine, L-ser: L-serine, L-met: L-methionine, L-cys: L-cystine, L-trp: L-tryptophan, L-lys: L-lysine, pmt-CoA: palmitoyl-Coenzyme A, ac-CoA: acetyl-Coenzyme A, orn: ornithine, Lkynr: L-kynurenine, glutCoA: glutaryl-Coenzyme A, hexdi-CoA: Hexadecanedioyl-Coenzyme A, 5mthf: 5-methyl tetrahydrofolate, 2oxoadp: 2-oxoadipate, 3bcrn: 3-hydroxybutyrylcarnitine, 3hadpac: 3-hydroxyadipate, 4aabutn: 4-acetamidobutanoate, 3hexdcrn: 3-hydroxyhexadecanoylcarnitine, fadh2: flavin adenine dinucleotide (reduced), fad:, flavin adenine dinucleotide (oxidized), q10h2: Ubiquinol-10, q10: Ubiquinone-10, 2obut: 2- oxobutanoate, h2o2: hydrogen peroxide. Abbreviations for metabolites and reactions use nomenclature from the BiGG database (http://bigg.ucsd.edu/).

Context-specific GSMMs of multiple cells were constructed for the simultaneous, integrated analysis of these data. Liver models were constructed using the metabolomics serum profiles from the experiments here, as well as transcriptome profiles of hepatocytes from *Ahr*^−/−^ and *Ahr*^+/+^ mice. MCF7 models were constructed using the transcriptome profiles of *AHR*-knockdown cells. Metabolic network simulations were carried out for *Ahr*^−/−^ and *Ahr*^+/+^ mice in both liver and MCF7, respectively and compared to identify reactions predicted to have altered flux states (Fig. 6A).

### *Ahr* knockout mouse liver transcriptomics integrated with serum metabolomics reveals role of AHR in ammonia and nitrogen moiety metabolism

We first focused on the liver transcriptomic data because of the existing data supporting the notion that AHR has a major impact on liver gene expression. The hepatocyte models identified 2192 reactions as being significantly differentially active (Supplemental Table S5). One hundred and two of these reactions were increased in the knockout relative to the wildtype model. Ammonia and nitrogen moiety elimination and metabolism dominated many of these reactions. Redox reactions were also implicated. Figure 6B highlights two different pathways for these processes, the phosphate dependent pathway and the NAD/NADH dependent pathway. The *Ahr*^+/+^ model has 7490 reactions and 4077 metabolites in contrast to the knockout model that has 7073 reactions and 3790 metabolites. There are 6143 shared reactions between the two models, with 1347 unique to the *Ahr*^+/+^ and 930 unique to the *Ahr*^−/−^. While the sizes of the models were similar, there are significant functional differences between the *Ahr*^+/+^ and *Ahr*^−/−^ liver models. For example, the largest *Ahr*^+/+^ reaction co-sets (see Materials and Methods), focusing on keratan sulfate synthesis and degradation as well as chondroitin sulfate metabolism, are not present in the *Ahr*^−/−^ models. Other differences in the co-sets involved fatty acid metabolism and intra-cellular transport (between cytosol, endoplasmic reticulum, and the peroxisome).

### Integration of *AHR* knockdown transcriptomics in cells with knockout metabolomics data emphasizes role of AHR in peroxisomal fatty acid degradation

In order to better appreciate the direct consequences of AHR function without a focus on liver-specific functions or inclusion of multiple cell populations, we then integrated transcriptomic data from the MCF7 cells siRNA *AHR* knockdown experiment with our serum metabolomics data to develop alternative GSMMs for both *Ahr*^−/−^ and *Ahr*^+/+^ mice. Metabolic network simulations were carried out for both genetic conditions and compared to identify reactions predicted to have altered flux states. 2169 reactions were significantly altered (Supplemental Table S6), and 168 of these reactions were increased in the knockout relative to wildtype condition. The key reactions and altered synthesis and degradation pathways are highlighted in Fig. 6C.

Importantly, the organelle-specific cellular processes and co-factor balances are highlighted to draw attention to the altered metabolic demands at multiple scales resulting from *Ahr* perturbation. Peroxisomal fatty acid degradation was increased for multiple fatty acids, resulting in elimination as organic acids (such as 3-hydroxyadipate) and generation of hydrogen peroxide. Increased cellular export of carnitine bound fatty acids was also observed via medium chain fatty acids as well as end products of tryptophan metabolism (via L-kynurenine and glutaryl-CoA). Collectively, increased elimination of carnitine bound fatty acids, ornithine end products, and other amino acid degradation pathways leading to 3-hydroxybutyrylcarnitine, are all suggestive of increased nitrogen elimination in the *Ahr*^−/−^ mice.

## Discussion

Taken together, the studies described provide new insights into the endogenous role of AHR in metabolism at multiple scales. We uncovered numerous biochemical pathways and functional clusters that were significantly altered in the serum of the *Ahr*^−/−^ mice. The loss of *Ahr* led to dozens of metabolites being elevated or decreased in the serum that were largely separable from one another by physicochemical properties, suggesting there may be a molecular basis for their alterations, presumably reflective of the set of transporters, enzymes, and other genes regulated by AHR. Among the altered metabolites, were three known AHR ligands: kynurenate, xanthurenate, and indolepropionate. Indolepropionate, the only metabolite known AHR ligand that was elevated in the *Ahr*^−/−^ mice, activated AHR in human and murine cell lines, with stronger activation in human than murine cells. Finally, we integrated existing transcriptomic data with our serum metabolomics data to generate metabolic reconstruction models using Recon3D that predicted novel roles for AHR in multiple pathways including, nitrogen elimination, peroxisomal/mitochondrial fatty oxidation, and other cellular processes (Fig. 6).

*Ahr*^−/−^ mice have been used to analyze transcriptional changes in multiple organs, but serum metabolomics data in these mice has been scarce. Here, our global metabolic profiling presented novel systemic metabolic roles for AHR and validated prior work. For example, it has been shown that AHR activation via dietary exposure to TCDD led to a significant decrease in total serum bile acids^34^. This and other related work complemented our results, as primary and secondary bile acid metabolism were among the most enriched subpathways for elevated metabolites, showing that activation of AHR and absence of *Ahr* have opposite effects on the circulating levels of bile acids^16^. In our metabolomics analysis, some of the other most impacted subpathways included fatty acid metabolism (acyl carnitine, hydroxy), pyrimidine metabolism, and glutathione metabolism for elevated metabolites, as well as other aspects of fatty acid metabolism (acyl choline), sphingomyelins, and lysophospholipid for decreased metabolites. To our knowledge, this is the first time AHR function has been associated with these metabolites, suggesting a key and underappreciated role in general physiology. However, it should be noted that these mice may have some developmental defects that may impact their “normal” endogenous metabolism, and some serum metabolite changes could be a result of this disrupted development^35^.

While the aforementioned metabolic pathway analysis helped in the interpretation of whole-scale changes, the individual metabolites were useful in understanding structural patterns of AHR regulation. The systemic changes in levels of circulating metabolites are likely due to the absence of *Ahr,* which leads to changes in the gene/protein expression patterns of AHR-expressing organs. It is possible that because AHR is known to regulate many transporters and enzymes with linked substrate specificities, certain physicochemical features collectively predispose a metabolite to altered metabolism/excretion. We therefore characterized the altered metabolites by their molecular descriptors and found that some properties were useful in distinguishing elevated compounds from decreased compounds. Understanding the structural similarities and differences between metabolites altered by loss of AHR can be useful in determining potential side effects from drugs or toxins that interact with AHR. For example, chronic treatment or exposure to a small molecule AHR inhibitor may partly reflect the changes in the knockout mice. This may be particularly important because it appears that many of the identified molecules have well-established signaling roles, such as the elevated bile acids which bind to multiple GPCRs and nuclear receptors^36,37^. Furthermore, many of the altered metabolites are also derived from the diet, such as cholines and tryptophan derivatives; knowledge of how their levels are affected by AHR could lead to improved dietary recommendations, particularly in the setting of the aberrant AHR-mediated metabolism believed to occur in liver and kidney disease^7^.

The logarithm of partition coefficients (molLogP), which is a measure of the hydrophobicity of compounds, was one of the physicochemical properties that best separated elevated and decreased metabolites (Fig. 4). The elevated metabolites in the *Ahr*^−/−^ mice on average had lower molLogP values than the decreased metabolites, indicating that decreased metabolites were more hydrophobic. The higher hydrophobicity values of decreased metabolites, when coupled with transcriptomic data from *AHR* knockdown and/or deletion experiments, suggests that AHR may play a role in adding hydrophilic groups to some of these metabolites to decrease their hydrophobicity and improve solubility for potential clearance. This is consistent with AHR’s role in the phase II metabolic pathway of glucuronidation, as shown previously by the role of AHR in activating UGT genes to glucuronidate endogenous compounds, such as estrogen and bile acids. AHR also exhibited a specialized xenobiotic response element for increasing transcription of *UGT1A3*^38^.

AHR is a ligand-activated transcription factor with a wide range of ligands and inhibitors (Supplementary Table S3). While many of these have been synthetically generated, recent work has uncovered a number of endogenous substrates^18,22,39^. Some tryptophan derivatives and eicosanoids measured with our global metabolomics approach are known to activate AHR at low concentrations. Previous work with indoxyl sulfate, a high affinity AHR ligand and uremic toxin, has shown that endogenous compounds can participate in feedback loops with ligand-activated receptors that modulate the levels of the activating ligand. Indoxyl sulfate is known to be transported into kidney cells through organic anion transporter 1 (OAT1/SLC22A6)^40,41^. It activates AHR in the kidney, which in turn, upregulates levels of OAT1 to increase clearance of extracellular indoxyl sulfate^30^. This feedback loop indicates that indoxyl sulfate can regulate its own systemic levels through activation of cell machinery via a ligand-activated transcription factor. Since the mice used here were not treated with any exogenous agents, we assumed that basal levels of AHR activation in the *Ahr*^+/+^ mice are the result of activation by these endogenous compounds and aimed to see if their levels were altered by the loss of AHR.

Although indoxyl sulfate was not significantly altered, we identified three endogenous metabolites (kynurenate, xanthurenate, and indolepropionate) that are known AHR ligands and were significantly altered in the serum of the knockout mouse. Kynurenate and xanthurenate were significantly decreased, while indolepropionate was elevated. We focused on indolepropionate, as the loss of AHR may lead to the diminished expression of proteins that aid in the metabolism/excretion of indolepropionate.

It is also possible that, just as AHR appears to play a key role in inter-organ communication via small molecules, AHR plays a role in inter-organismal communication between the host and the gut microbiome. Thus, loss of *Ahr* may alter the composition of the gut microbiome, which produces indolepropionate and other metabolites. Indolepropionate regulation is particularly important to understand as it has been shown to have several important physiological roles, suggesting that its regulation is crucial. For example, indolepropionate is involved in the strengthening of the intestinal barrier via PXR activation, as well as radical scavenging and neuroprotectivity^42,43^.In addition to indolepropionate and secondary bile acids (which are also generated by the gut microbiome), we also noted that p-cresol sulfate and p-cresol glucuronide were significantly elevated. The production of these compounds is associated with the gut microbiome^44^ While we did not explore the connection between the gut microbiome and host in this study, it is important to understand the many levels of coordination that determine the circulating levels of certain metabolites. Indeed, some studies have indicated a gut microbiome dependent modulation of AHR^45,46^.

Modeling AHR-mediated metabolism is difficult because of its expression in various tissues, as well as its distinct role in each tissue and responses to different ligands. To best determine how AHR might affect intracellular activity in the context of its effect on systemic metabolism, we integrated cellular transcriptomics with extracellular metabolomics to develop a constrained metabolic reconstruction of multiple scales of metabolism using Recon3D. While genome-scale metabolic reconstructions continue to advance and aim to provide a broader view of the effects of gene perturbation in metabolism at many scales not usually considered by conventional pathway analysis, they do not yet fully capture many aspects of metabolism. That said, Recon3D, is currently the most comprehensive version, and it, along with previous versions, have been useful for providing insights into altered metabolism in xenobiotic transporter knockout mice^31–33,47^. Nuclear receptor signaling is not well-defined in Recon3D; thus, instead of removing a single gene, we used the data from AHR-related experiments to approximate its systemic role. The metabolic reconstruction model revealed the importance of AHR in nitrogen elimination in the hepatocyte model and peroxisomal fatty acid oxidation in the MCF7 model. Peroxisomes serve critical roles in processes such as beta oxidation, bile acid synthesis, and reactive oxygen species generation^48,49^. Previous work has shown that inhibition of AHR in liver lead to steatosis, potentially via AHR inhibition of peroxisomal beta oxidation of fatty acids^50^ or decreased expression of a transporter like OAT1, which contributes to lipid metabolism^31^. Acetyl-CoA Acetyltransferase, the most altered reaction in our MCF7 metabolic reconstruction, is an enzyme involved in the final step of mitochondrial beta-oxidation and is critical for ketone body metabolism. Among the other pathways implicated in our model was the kynurenine clearance pathway, which supports a connection between AHR and tryptophan metabolism; it is worth noting that kynurenine activates human and murine AHR at nearly the same level (Supplementary Figure S1). To our knowledge, this is the first mammalian genome-scale metabolic reconstruction that uses multi-omic data to describe the function of a transcriptional regulator, most of which are absent in metabolic reconstructions.

Our results also align with the Remote Sensing and Signaling Theory (RSST), which describes the role of multi-specific proteins in the collective handling of endogenous metabolites in the service of homeostasis, among other things^5,6,51^. While the RSST primarily focuses on drug transporters and drug metabolizing enzymes in organ crosstalk (e.g., gut-liver-kidney) and inter-organism communication (e.g., microbes-host) via small molecules, there is an important and underappreciated role for the proteins that regulate the expression of multi-specific proteins. These nuclear receptors and ligand activated nuclear receptors, which can have multiple, distinct ligands, were included in a proposed Remote Sensing and Signaling Network in an effort to better capture the full range of proteins involved in small molecule handling^27^. We further explored this here by analyzing the functional consequences of the loss of a ligand activated transcription factor on circulating metabolites, confirming that AHR and potentially other nuclear receptors and ligand activated transcriptional factors play a critical role in endogenous metabolism through the modulation of small molecule metabolism, which was partly characterized by our chemoinformatic analysis. By integrating these metabolomic data with transcriptomic data, we were able to implicate AHR in cellular and organelle metabolism. Taken together, we have demonstrated the different metabolic roles that AHR plays at multiple scales (organism, organ, organelle) using a multi-omic approach and support its importance in the regulation of organ crosstalk via small molecules.

## Methods

### Animals

C57BL6/J wildtype and C57B6.129-*Ahr*^*tm1Bra*^/J (*Ahr*^−/−^) mice were originally obtained from The Jackson Laboratory (Bar Harbor, ME.) and as a gift, respectively. They were, subsequently bred in-house^52^. Mice were weaned onto and maintained with ad libitum access to water and standard rodent chow [PicoLab Rodent Diet 20 #5053] (St. Louis, MO.) in a specific pathogen-free vivarium (The Pennsylvania State University). 8–10-week-old female *Ahr *^−/−^ (n = 7) and wildtype mice (n = 5) were euthanized by carbon dioxide asphyxiation (flow-rate 1L/min), and whole blood from the portal vein was collected into Becton Dickinson Microtainer serum separator tubes (Franklin Lakes, NJ.). After 0.5 h coagulation, samples were centrifuged (1200 × *g*, 15 min, 4 °C), and sera were transferred to 1.5 ml tubes and snap-frozen in liquid nitrogen. All samples were stored at − 80 °C until analysis. All animal studies were conducted in accordance with NIH guidelines and under the auspices of animal use protocols approved by the Pennsylvania State University Institutional Animal Care and Use Committee. All experiments were carried out in compliance with ARRIVE guidelines.

### Metabolomics data analysis

Serum samples were sent to Metabolon (Durham, NC) for global metabolic profiling analysis. Intensity values were normalized to volume, log transformed, and missing values were imputed with the lowest measured value for each metabolite. Welch’s t-test was used to determine statistical significance between groups, and FDR-adjusted *p*-values were calculated. Heatmaps were generated using the ComplexHeatmap in R software environment^53^.

### Calculation of physicochemical properties and chemoinformatics analysis

SMILES sequences were obtained from the Pubchem IDs of each metabolite. The ChemRICH web-based tool was used to perform metabolite set enrichment analysis based on chemical similarity. The functional clusters were then ranked by statistical significance using the Kolmogorov-Smirnov test^28^. One dimensional properties for each metabolite were calculated using ICM Molsoft Pro (San Diego, CA). Morgan fingerprints for each metabolite were calculated using RDkit. Similarity between molecules was determined using the Tanimoto similarity index in RDkit. Visualizations were performed using the Seaborn package in Python 3.8.

### Transcriptomic data analysis

Transcriptomic datasets for AHR activation/inhibition were acquired from the Gene Expression Omnibus (GEO) database and were analyzed for differentially expressed genes using Bowtie, SAMtools, bioMart, and DESeq in an R environment. Gene ontology enrichment was performed using the Enrichr web tool with significantly altered genes as inputs.

### Cell lines and culture conditions

HepG2 40/6 and Hepa1.1 reporter cell lines were obtained and cultured as previously reported^54^.

### Cell-based reporter assay

Hepa1.1 and HepG2 40/6 cells were seeded into 12-well tissue culture plates (Corning Inc., Corning, NY, USA) at a density of 5 × 10^4^ cells/well and allowed to recover for 24 h or 48 h, respectively. Cells were treated with increasing amounts of kynurenine (Kyn), indole-3-propionic acid (IPA), xanthurenic acid (XA), and TCDD (10 nM final concentration) in DMSO (0.2% final concentration in cell culture). After a 4 h incubation period, cells were thoroughly washed with PBS prior to addition of a lysis buffer composed of 25 mM Tris–phosphate (pH 7.8), 2 mM dithiothreitol, 20 mM 1,2-cyclohexanedinitrilotetraacetic acid, 10% (v/v) glycerol and 1% (v/v) Triton X-100, and stored at − 80 °C until analysis. The thawed lysate was assayed for luciferase activity using the Promega Luciferase assay system (Madison, WI, USA), as per the manufacturer’s instructions, and a TD-20E luminometer (Turner Designs Inc., San Jose, CA, USA). Data were analyzed and plotted using Prism 9 (Graphpad Software, San Diego, CA, USA). Response values were normalized using DMSO and TCDD treatments to determine minimal and maximal activation. Dose–response curves were fitted using nonlinear, least squares fit, and treatments were statistically compared using a sum-of-squares F test.

### Metabolic reconstructions

We used genome scale reconstruction (Recon3D) to carry out a systemic functional pathway analysis^55^. Since AHR is generally expressed in multiple tissues, and serum metabolomics reflect interactions with multiple tissues and organs, we used the general reconstruction as the context to interpret changes in gene expression and serum metabolomics between the knockout and wildtype mice. In general, reaction fluxes do not correlate with gene expression in a quantitative manner; however, present/absent calls are a more biologically consistent approach, and thus we used the GiMME present/absent calls to interpret the upregulated and downregulated genes of interest by comparing wild type and AHR knockout cells^56^. Hepatocyte wildtype and knockout models were constructed based on calculation of differentially expressed genes with significance cutoff of *p* < 0.05 with Benjamini-Hochberg false discovery rate corrections^57^. We also performed reconstructions for the MCF7 cell line data set and used the upregulated genes to be simulated as “absent” genes in the wildtype condition and the downregulated genes to be “absent” in the knockdown condition^58^.The metabolomic data and constraints were then incorporated as described below.

In order to minimize non-specific calculations, only the set of measured metabolites (in addition to ions, water, and oxygen) were permitted to be taken up by the models. Flux Variability Analysis (FVA) was then performed to identify the subset of metabolites that were either only taken up or only secreted. This was done to approximate either net production or consumption of a metabolite across the entire organism. Metabolomic data constraints test with FVA was used to classify metabolites into three groups, those that can only be secreted, those that can only be taken up, and those that can be secreted or taken up. Interpretation of the metabolomic fold change measurements (*Ahr*^−/−^ relative to *Ahr*^+/+^) were as follows:

- If *Ahr*^−/−^/*Ahr*^+/+^ > 1 and the metabolite could only be secreted, then the metabolite exchange was constrained with a lower bound set to 50% of the maximum;
- If *Ahr*^−/−^/*Ahr*^+/+^ > 1 and the metabolite could only be taken up, then the metabolite exchange was constrained with a lower bound that was 50% of the minimum;
- If *Ahr*^−/−^/*Ahr*^+/+^ < 1 and the metabolite could only be secreted, then the metabolite exchange was constrained with an upper bound that was 50% of the maximum;
- If *Ahr*^−/−^/*Ahr*^+/+^ < 1 and the metabolite could only be taken up, then the metabolite exchange was constrained with upper bound that was 50% of the minimum.

GiMME was used to construct the *Ahr*^−/−^ and *Ahr*^+/+^ models^56^. Simultaneously the metabolomic data changes were incorporated through adjusting the corresponding exchange reaction constraints. Subsequent simulations were performed for the two models. Differential mean flux states were then computed from the normalized sampled feasible flux states, requiring the following conditions: *p* < 0.00001 for Kolmogorov-Smirnov test as well as *F*-test, with at least > 5-fold change (or < 0.2 fold change). One hundred and six of the significantly altered metabolites mapped to model exchange reactions; of these, 92 were constrained (based upon uni-directional uptake or secretion). Correlated reaction sets were calculated for correlation coefficients greater than or equal to 0.975 (or R-squared > 0.95). Model modifications and simulations were carried out with using CobraPy and the CobraToolbox with the Gurobi Optimizer (v 8.0)^59,60^. The network pathway map was created using Escher^61^.

## Supplementary Information

Supplementary Information 1.

Supplementary Information 2.

Supplementary Information 3.

Supplementary Information 4.

Supplementary Information 5.

Supplementary Information 6.

Supplementary Information 7.

## Data Availability

The data presented in this study are available in the Supplementary Information.
