# Supplementary material for: AHR is a master regulator of diverse pathways in endogenous metabolism
Source: Sci Rep. 2022 Oct 5;12:16625. doi: 10.1038/s41598-022-20572-2 (PMC9534852; doi:10.1038/s41598-022-20572-2)
Supplement: Supplementary file 7 — Supplementary Information 7. [file 41598_2022_20572_MOESM7_ESM.pdf]

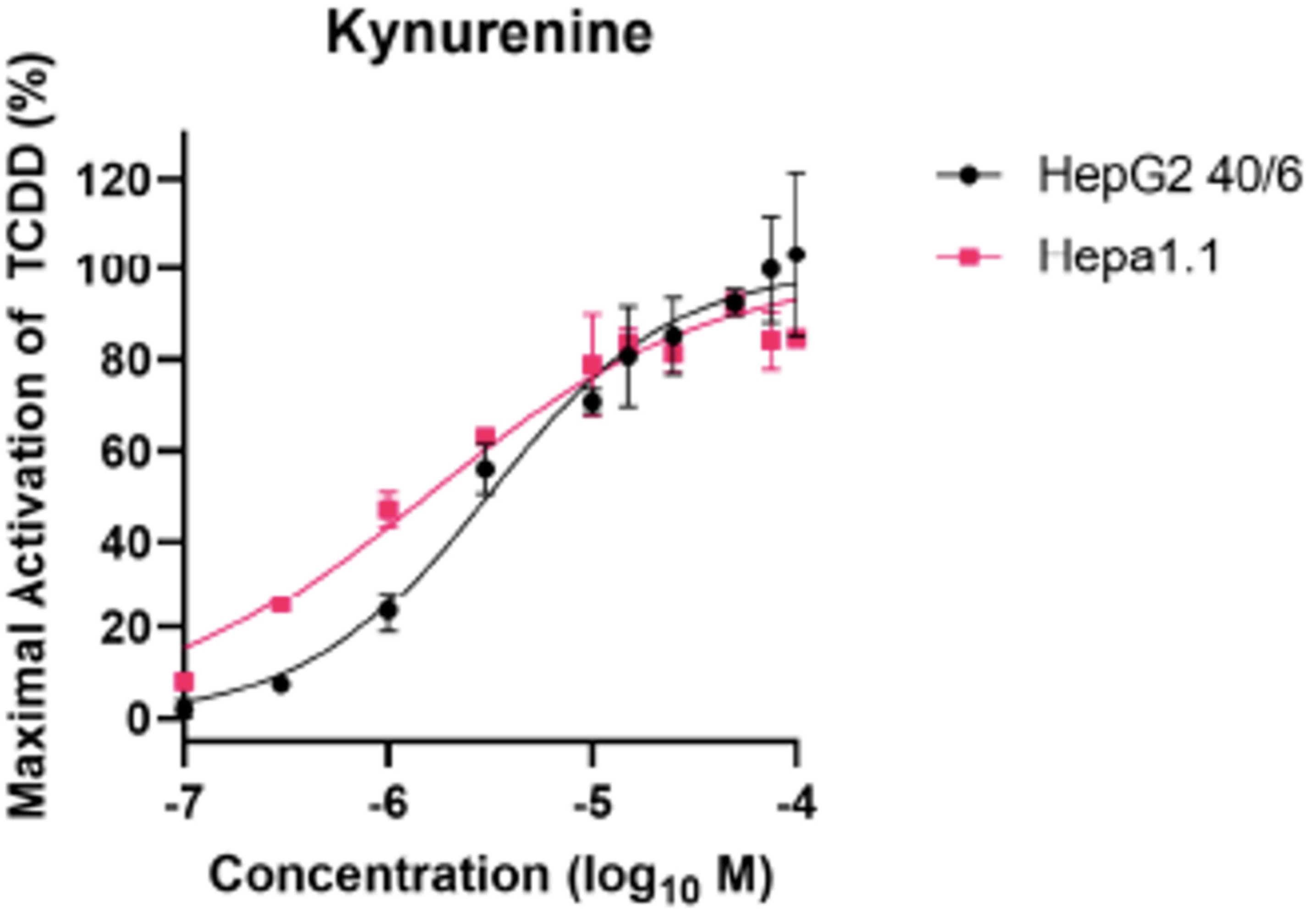

Supplementary Figure S1: Kynurenine was assessed for its ability to activate both human and murine AHR at ten biologically relevant doses (100 nM, 300 nM, 1, 3, 10, 15, 25, 50, 75, 100 µM final concentrations). TCDD (10 nM final concentration) was used to determine maximal activation of AHR, and the presented values were normalized as a percentage of TCDD activation. Each data point represents the mean ± S.D. of three biological replicates.
